# A Comprehensive Comparative Analysis of the Histomorphological Features of *ALK*-Rearranged Lung Adenocarcinoma Based on Driver Oncogene Mutations: Frequent Expression of Epithelial-Mesenchymal Transition Markers than Other Genotype

**DOI:** 10.1371/journal.pone.0076999

**Published:** 2013-10-23

**Authors:** Hyojin Kim, Se Jin Jang, Doo Hyun Chung, Seol Bong Yoo, Pingli Sun, Yan Jin, Kyung Han Nam, Jin-Ho Paik, Jin-Haeng Chung

**Affiliations:** 1 Department of Pathology, Seoul National University Bundang Hospital, Seongnam, Republic of Korea; 2 Department of Pathology, Seoul National University College of Medicine, Seoul, Republic of Korea; 3 Department of pathology, Asan Medical Center, University of Ulsan College of Medicine, Seoul, Republic of Korea; 4 Department of Pathology, Presbyterian Medical Center, Jeonju, Republic of Korea; Consiglio Nazionale delle Ricerche (CNR), Italy

## Abstract

Molecular classification of lung cancer correlates well with histomorphological features. However, specific histomorphological features that differentiate anaplastic lymphoma kinase (*ALK*)-rearranged tumors from *ALK*-negative tumors have not been fully evaluated. Eighty *ALK-*rearranged and 213 *ALK*-negative (91 epidermal growth factor receptor*-*mutated; 29 *K-ras-*mutated; 93 triple-negative) resected lung adenocarcinomas were analyzed for several histomorphological parameters and histological subtype. *ALK*-rearranged tumors were associated with younger age at presentation, frequent nodal metastasis, and higher stage of disease at diagnosis. *ALK*-rearranged tumors were more likely to show a solid predominant pattern than *ALK*-negative tumors (43.8%; 35/80; *p*<0.001). Unlike *ALK*-negative tumors, a lepidic predominant pattern was not observed in *ALK*-rearranged tumors (*p*<0.001). In multivariate analysis, the most significant morphological features that distinguished *ALK*-rearranged tumors from *ALK*-negative tumors were cribriform formation (odds ratio [OR], 3.253; *p = *0.028), presence of mucin-containing cells (OR, 4.899; *p = *0.008), close relationship to adjacent bronchioles (OR, 5.361; *p = *0.001), presence of psammoma bodies (OR, 4.026; *p = *0.002), and a solid predominant pattern (OR, 13.685; *p = *0.023). *ALK*-rearranged tumors exhibited invasive histomorphological features, aggressive behavior and frequent expression of epithelial-mesenchymal transition markers (loss of E-cadherin and expression of vimentin) compared with other genotype (*p = *0.015). Spatial proximity between bronchus and *ALK-*rearranged tumors and frequent solid histologic subtype with p63 expression may cause diagnostic difficulties to differentiate squamous cell carcinoma in the small biopsy, whereas p40 was rarely expressed in ALK-rearranged adenocarcinoma. Knowledge of these features may improve the diagnostic accuracy and lead to a better understanding of the characteristic behavior of *ALK*-rearranged tumors.

## Introduction

Adenocarcinoma of the lung is the most common histological type of primary lung cancer [1] and is a heterogeneous tumor with diverse molecular, clinical, and pathological characteristics. Identification of molecular driver mutations and their therapeutic implications in lung adenocarcinoma have become an important area of research as evidenced by the abundance of genomic, mutational, and proteomic profiling studies. [2], [3] Many studies have shown correlations between morphological features and molecular alterations in lung adenocarcinoma. Previous reports have investigated the association between epidermal growth factor receptor (*EGFR*) mutations and specific histological subtypes of adenocarcinoma such as lepidic (formerly known as nonmucinous bronchioloalveolar carcinoma), papillary, and micropapillary patterns.[4]–[7] In contrast, *KRAS* mutation status has been shown to be significantly associated with solid and invasive mucinous adenocarcinoma subtypes. [8], [9] Therefore, these findings raise the fundamental question of whether morphological features reflect the presence of molecular alterations.

The presence of anaplastic lymphoma kinase (*ALK*) gene rearrangement in lung adenocarcinomas is the best predictor of response to crizotinib, an ALK tyrosine kinase inhibitor. [10], [11] Fluorescence *in situ* hybridization (FISH) has been established as the gold standard method for the detection of *ALK* rearrangement in lung adenocarcinoma. The Food and Drug Administration approved crizotinib with a companion diagnostic FISH test for *ALK*-rearranged non-small cell lung cancer (NSCLC). Several studies have investigated the predictive value of pathological and morphological features in detecting *ALK*-rearranged tumors; however, the results of these studies have been inconsistent because of the limited number of *ALK*-rearranged tumors. [12]–[16] Solid signet-ring cell subtypes and cribriform pattern have been associated with *ALK* rearrangement in lung adenocarcinoma. [12], [15] A few studies have reported a positive histological correlation with *ALK* rearrangement in lung adenocarcinoma using the new International Association for the Study of Lung Cancer, American Thoracic Society and European Respiratory Society (IASLC/ATS/ERS) classification that was published in 2011. [16], [17] However, the comparative analysis of these histomorphological features and subtypes of *ALK*-rearranged lung adenocarcinoma based on driver oncogene mutations has not been clearly established in lung adenocarcinoma.

The aim of this study was 1) to evaluate the clinicopathological and histological features of 80 cases of *ALK*-rearranged resected lung adenocarcinomas, 2) to compare these features with those of *ALK*-negative tumors expressing well-known driver mutations associated with lung adenocarcinoma, and 3) to investigate the correlation between molecular subtype and histological features of lung adenocarcinoma based on the new IASLC/ATS/ERS classification.

## Materials and Methods

### Case Selection

This study was approved by the Institutional Review Board at Seoul National University Bundang Hospital. Written informed consent was specifically waived by the approving IRB in this study. A total of 80 surgically resected lung adenocarcinoma specimens harboring *ALK*-rearrangement were retrieved from the files of Seoul National University Affiliated Hospitals and the Asian Medical Center between January 2004 and June 2011. In addition, 213 *ALK*-negative resected adenocarcinoma specimens obtained from patients diagnosed between March 2009 and March 2010 were included in the study. Of the 213 *ALK*-negative tumors, 91 were *EGFR*-mutated, 29 were *K-ras*-mutated, and 93 were triple-negative (TN; wild-type *EGFR, K-ras,* and *ALK*). Patients who had a previous history of cancer, presurgical chemotherapy or radiotherapy were excluded. All cases were classified according to the seventh edition of the Union for International Cancer Control/American Joint Committee on Cancer TNM classification. [18] Clinicopathological information was obtained from the medical records and pathology reports.

### Histological Analysis

All resected specimens were fixed with formalin and stained with hematoxylin and eosin (H&E). All slides, including those of normal lung tissue, were carefully reviewed by 2 of the authors (H.K. and J.H.C.). An average of 8.9 slides (range: 1–14 slides) from each case was reviewed. Recent reports have demonstrated a strong association of extracellular mucin and cribriform pattern with *ALK*-rearranged tumors. [19] Therefore, the presence of extracellular mucin and cribriform architecture and the proportion of mucin-containing cells were evaluated in *ALK*-rearranged and *ALK*-negative tumors. *ALK*-rearranged tumors tended to be centrally located near the bronchus; therefore, the anatomic relationship between the tumor and the bronchi was investigated. The following histological parameters were evaluated: tumor location in relation to the bronchus; tumor invasion to the bronchus; alterations in bronchial epithelial cells located adjacent to the tumor; psammomatous calcifications; cholesterol cleft; tumor size; pathological stage; and visceral pleural, vascular, and lymphatic invasion. Adenocarcinoma *in situ* and minimally invasive adenocarcinoma cases were excluded from the study. All invasive adenocarcinomas were categorized as lepidic, papillary, acinar, micropapillary, solid predominant, and invasive mucinous adenocarcinoma (IMA) according to the IASLC/ATS/ERS classification [17].

### Detection of *ALK* Gene Rearrangement

*ALK* rearrangement in formalin-fixed, paraffin-embedded tumor tissues was detected by FISH analysis using a break-apart probe specific for the *ALK* locus (Vysis LSI ALK dual-color, break-apart rearrangement probe; Abbott Molecular, Abbott Park, IL, USA). FISH-positive cases were defined as those with >15% split signals or an isolated red signal in tumor cells as described previously [20]–[22].

### Detection of *EGFR* and *K-ras* Mutations

Genomic DNA was extracted from paraffin-embedded tissues. After deparaffinization with xylene, tissue sections were stained with H&E, and target lesions were selectively dissected to minimize contamination with normal tissue. Genomic DNA was isolated using the QIAamp DNA Mini Kit (Qiagen, Hilden, Germany) according to the manufacturer’s instructions. *EGFR* mutations at exons 18–21 and *K-ras* mutations at codons 12, 13, and 61 were analyzed by nested polymerase chain reaction (PCR) and direct DNA sequencing as described previously. [23] PCR products were processed using the Big Dye Terminator v3.1 Cycle Sequencing Kit (Applied Biosystems, Foster, CA, USA), and sequence data were generated using the ABI PRISM 3100 DNA Analyzer (Applied Biosystems).

### Immunohistochemistry

Immunohistochemistry was performed on tissue microarray sections. Four-micrometer-thick sections were transferred to poly-l-lysine-coated glass slides and incubated in a dry oven at 60°C for 1 h. The sections were then dewaxed in xylene (3 changes), rehydrated in a descending series of graded ethanol concentrations, and rinsed in Tris-buffered saline (TBS; pH 7.4). The endogenous peroxidase activity was blocked using 5% hydrogen peroxide in methanol for 15 min at 37°C. For antigen retrieval, the slides were placed in citrate buffer (10% citrate buffer stock in distilled water, pH 6.0) and microwaved for 10 min. Nonspecific staining was blocked using 1% horse serum in TBS (pH 7.4) for 3 min. The following primary antibodies were used: mucin-1 (MUC-1; 1∶100; Ma695; Novocastra), surfactant protein (SP)-A (1∶200; PE10; Dako), SP-B (1∶100; SPB01; Neomarker), SP-C (1∶100; FL-197; Santa Cruz Biotechnology), thyroid transcription factor-1 (TTF-1; 1∶100; 8G7G3/1; Dako), p63 (1∶100; 4A4; Zeta Corporation), p40 (1∶200; rabbitpoly; Biocare), E-cadherin (1∶150; SPM471; Thermo Fisher Scientific) and vimentin (1∶100; V9; Dako). Immunostaining was developed using an avidin–biotin–peroxidase complex (Universal Elite ABC Kit; PK-6200; Vectastain, Burlingame, CA, USA) and diaminobenzidine tetrahydrochloride solution (HK153-5K; Biogenex, San Ramon, CA, USA). Positive controls (samples with known reactivity for the antibody) and negative controls (omission of the primary antibody) were included in each assay.

MUC-1, SP-A, SP-B, and SP-C immunostaining was scored as the percentage of positively stained neoplastic cells: 0, no positively stained cells; 1+, 0–24% positively stained cells; 2+, 25–49% positively stained cells; 3+, 50–74% positively stained cells; and 4+, 75–100% positively stained cells. Immunostaining present in the cytoplasm, cell apex (luminal surface), and associated secretory products (luminal contents) was evaluated separately. Cells were considered positive when at least 1 of these components stained positively. Cytoplasmic, luminal surface, or luminal immunostaining in ≥25% of tumor cells (score ≥2) was considered positive for MUC-1, SP-A, SP-B, and SP-C. [24] For TTF and p63 nuclear immunostaining in >10% of tumor cells was considered positive. [14] p40 immunostaining was scored multiplying the percentage of immunoreactive cells (0% to 100%) by the immunostaining intensity (low = 1 vs strong = 2, according to internal controls). [25] E-cadherin and vimentin immunostaining was scored using a semiquantitative approach; the percentage of positive tumor cells (0–100%) was multiplied by the staining intensity (0, negative; 1, weak; 2, moderate; 3, strong) to generate a total score ranging from 0–300 for each sample. Samples with a score of 0–100 and 101–300 were classified as negative and positive, respectively [26].

### Statistical Analysis

Pearson’s chi-square test, Fisher’s exact test, and one-way analysis of variance were used to evaluate the association of clinicopathological and histological variables with lung adenocarcinoma genotype. Multivariate logistic regression analysis was used to determine the most significant morphological features associated with *ALK*-rearranged tumors. All statistical tests were two-sided, and a *p* value <0.05 was considered statistically significant. All analyses were performed using SPSS version 18.0 (SPSS Inc., Chicago, IL, USA).

## Results

### Patient Characteristics

The clinicopathological characteristics of the 293 patients are listed in Table 1. Of the 293 patients, 147 (50.2%) were men and 146 (49.8%) were women. The median age was 61.97 years (range, 30 to 83 years). Of the 293 patients, 175 patients (59.7%) were never-smokers and 118 (40.3%) were smokers (68, ex-smokers; 50, current-smokers). Acinar predominant (44.7%) was the most common histological subtype, followed by solid predominant (21.2%), papillary predominant (20.8%), lepidic predominant (5.5%), micropapillary predominant (4.4%), and IMA (3.4%). According to pathological stage, 79%, 44%, 62%, and 8% of the cases were p-stage I, stage II, stage III, and stage IV, respectively.

**Table 1:** Clinicopathological characteristics of 293 patients with lung adenocarcinoma.

Characteristics		Patients No. (%)
**Sex**	Male	147 (50.2%)
	Female	146 (49.8%)
**Age (years)**	Median (Range)	61.97 (30–83)
**Smoking history**	Never	175 (59.7%)
	Ex-smoker	68 (23.2%)
	Current smoker	50 (17.1%)
**Tumor size(cm)**	Mean (Range)	3.13 (0.7–13.5)
**Nodal metastasis**		96 (32.8%)
**Histologic subtype**	Lepidic predominant	16 (5.5%)
	Papillary predominant	61 (20.8%)
	Acinar predominant	131 (44.7%)
	Solid predominant	62 (21.2%)
	Micropapillary predominant	13 (4.4%)
	Invasive mucinous	10 (3.4%)
**pTNM stage**	I	179 (61.1%)
	II	44 (15.0%)
	III	62 (21.2%)
	IV	8 (2.7%)
**Total**		293 (100%)

### Clinicopathological Findings

#### Comparison of the clinicopathological features between ALK-rearranged and ALK-negative tumors

The clinicopathological features of lung adenocarcinoma according to mutation status are shown in Table 2. *ALK*-rearranged tumors were significantly associated with younger age at presentation, frequent nodal metastasis, and higher stage of disease at diagnosis when compared with *ALK-*negative tumors (*p*<0.001). The median age of patients with *ALK*-rearranged tumors was 55.82 years, whereas the median age of patients with *EGFR*-mutated, *K-ras*-mutated, and TN tumors was 63.29, 64.86, and 65.1 years, respectively. Of the 80 patients with *ALK*-rearranged tumors, 59.0% (46/80) showed nodal metastasis at diagnosis and 50% (40/80) presented with advanced stage (III or IV) disease. Patient gender, smoking status, and tumor size were not significantly associated with *ALK*-rearranged tumors. The frequency of *ALK-*rearranged (55%; 44/80) and *EGFR*-mutated (68.0%; 61/91) tumors was significantly higher (*p*<0.001) in female patients than that of *K-ras*-mutated (31.0%; 9/29) and TN (34.4%; 32/93) tumors. *ALK*-rearranged (70%; 56/80) and *EGFR*-mutated (70.3%; 64/91) tumors were significantly higher (*p*<0.001) in never-smokers than *K-ras*-mutated (41.4%; 12/29) and TN (46.2%; 43/93) tumors. Tumor size was not significantly different between *ALK*-rearranged and *ALK*-negative tumors.

**Table 2:** Clinicopathological characteristics of patients based on driver mutation status.

	*ALK*+(n = 80)	*EGFR*+(n = 91)	*K-ras*+(n = 29)	TN(n = 93)	*p* value
**Sex**					***p*** **<0.001**
M	36(45%)	30(33.0%)	20(69.0%)	61(65.6%)	
F	44(55%)	61(68.0%)	9(31.0%)	32(34.4%)	
**Age (years)**					***p*** **<0.001**
Median	55.82	63.29	64.86	65.1	
**Smoking history**					***p*** **<0.001**
Never	56(70.0%)	64(70.3%)	12(41.4%)	43(46.2%)	
ex-smoker	10(12.5%)	16(17.6%)	10(34.5%)	32(34.4%)	
current smoker	14(17.5%)	11(12.1%)	7(24.1%)	18(19.4%)	
**Tumor size(cm)**	3.25±1.85	2.74±1.85	3.52±2.73	3.11±1.75	*p*>0.5
**Nodal metastasis**	46(59.0%)	19(20.9%)	6(24.0%)	25(26.9%)	***p*** **<0.001**
**pTNM stage**					***p*** **<0.001**
I	29(36.2%)	71(78.0%)	18(62.1%)	61(65.6%)	
II	11(13.8%)	11(12.1%)	9(31.0%)	13(14.0%)	
III	32(40.0%)	9(9.9%)	2(6.9%)	19(20.4%)	
IV	8(10.0%)	0	0	0	

Abbreviations: TN: triple negative.

### Histomorphological Findings

#### Comparison of the morphological features between ALK-rearranged and ALK-negative tumors

**Figure 1:**
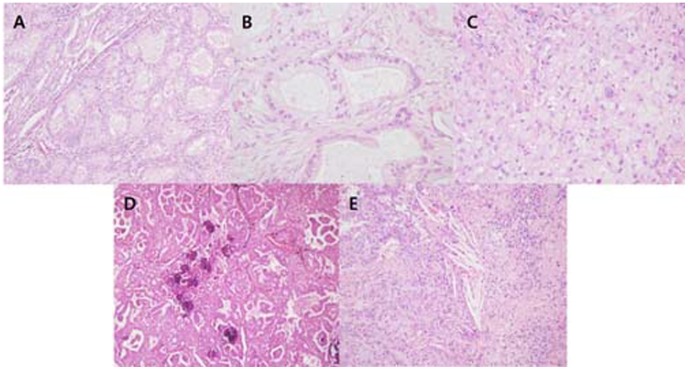
Histological characteristics of *ALK*-rearranged tumors. A, cribriform formation; B, mucin-containing goblet cells; C, mucin-containing signet-ring cell; D, psammoma body; E, cholesterol cleft.

**Figure 2:**
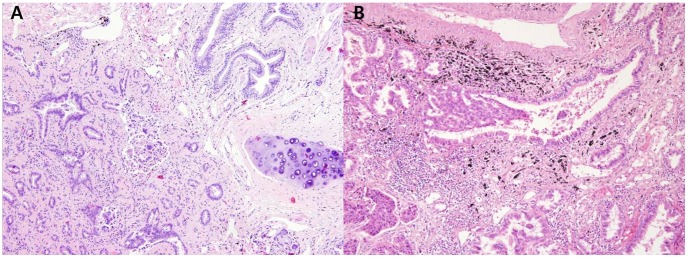
Relationship of *ALK*-rearranged tumors with the bronchiole. A, Tumor gland that resembles the adjacent segmental bronchial gland in morphology; B, Tumor cell infiltration of the adjacent bronchiolar epithelium.

Focal cribriform formation was present in 40.0% (32/80) of *ALK*-rearranged tumors (Figure 1A). Significant extracellular mucin and mucin-containing cells, including goblet cells (Figure 1B) and signet-ring cells (Figure 1C), were observed in 57.5% (46/80) and 62.5% (50/80) of *ALK*-rearranged tumors, respectively. Three *ALK*-rearranged tumor cases showed morphology similar to IMA, including a predominant lepidic pattern of goblet cell proliferation with abundant extracellular mucin. We noted that mucin containing tumor cells resemble the non-neoplastic goblet cells in segmental bronchus or bronchiole level. Several tumor glands resembled adjacent bronchial gland in morphology (Figure 2A). In some *ALK*-rearranged cases, tumor cells invaded the adjacent bronchiolar epithelium and showed the appearance of ‘budding off’ of small epithelial cell clusters into the lumen (Figure 2B). Furthermore, flat atypical epithelial lesions that resembled adjacent tumor cells infiltrated the non-neoplastic bronchial epithelium. This close anatomic relationship with the bronchus was observed in 86.3% (69/80) of *ALK*-rearranged tumors. Psammoma bodies (Figure 1D) and cholesterol clefts (Figure 1E) were observed in 56.4% (44/80) and 40.5% (32/80) of *ALK*-rearranged tumors, respectively. All the morphological characteristics described above were less evident in *ALK*-negative tumors (*p*<0.001).

#### Correlation between molecular subtype and histological features of lung adenocarcinoma based on the new IASLC/ATS/ERS classification

The histomorphological features of the 4 molecular subtypes based on the new IASLC/ATS/ERS lung adenocarcinoma classification are summarized in Table 3. *ALK*-rearranged tumors showed various histological patterns. The frequency of a solid predominant pattern was significantly higher in *ALK*-rearranged tumors than in *ALK*-negative tumors (43.8%; 35/80; *p*<0.001). In contrast, the frequency of acinar predominant histology was significantly lower in *ALK*-rearranged tumors than in *ALK*-negative tumors (28.7%; 23/80; *p*<0.001). In contrast to *ALK*-negative tumors, lepidic predominant histology was not observed in *ALK*-rearranged tumors (*p = *0.003). For *EGFR*-mutated and TN tumors, acinar predominant pattern was the most frequently observed histology (*EGFR*-mutated: 58.2%, 53/91; TN: 40.9%), followed by papillary predominant pattern (*EGFR*-mutated: 24.2%, 22/91; TN: 20.4%, 19/93). Acinar predominant was the most frequently observed pattern in *K-ras*-mutated tumors (58.6%; 17/29), followed by solid predominant (17.2%; 5/29). IMA was rarely observed in *ALK*-rearranged (3.7%; 3/80), *EGFR*-mutated (2.2%; 2/91), and TN (5.4%; 5/93) tumors. Histological patterns are assessed semiquantitatively in 5% increments in the new IASLC/ATS/ERS classification. Therefore, all visible patterns over 5% were recorded, and tumors were classified according to the presence of any histological subtype. The frequency of at least 5% solid pattern was significantly higher in *ALK*-rearranged tumors than in *ALK*-negative tumors (67.5%, 19.8%, 37.9%, and 26.9% of *ALK*-rearranged, *EGFR*-mutated, *K-ras*-mutated, and TN tumors, respectively; *p*<0.001). Acinar and lepidic patterns were less frequently observed in *ALK*-rearranged tumors than in *ALK*-negative tumors (acinar, 53.8%; lepidic, 12.5%; *p*<0.001). The frequency of at least 5% acinar pattern (90.1%) and at least 5% lepidic pattern (57.1%) was significantly higher in *EGFR*-mutated tumors when compared with the other molecular subtypes (*p*<0.001). *EGFR*-mutated tumors had the lowest frequency of solid pattern among the 4 molecular subtypes (19.8%; *p*<0.001).

**Table 3:** Histomorphological characteristics of lung adenocarcinoma based on driver mutation status.

	*ALK*+(n = 80)	*EGFR*+(n = 91)	*K-ras*+(n = 29)	TN(n = 93)	*p* value
**Histologic features**					
Cribriform formation	32(40.0%)	6(6.6%)	1(3.4%)	18(19.3%)	***p*** **<0.001**
Extracellular mucin	46(57.5%)	16(17.6%)	5(17.2%)	26(27.9%)	***p*** **<0.001**
Mucin-containing cells	50(62.5%)	18(19.8%)	5(17.2%)	27(29.0%)	***p*** **<0.001**
Relation with bronchus	69(86.3%)	39(42.8%)	12(41.4%)	44(47.3%)	***p*** **<0.001**
** **bronchiole	52(65.0%)	34(37.4%)	11(37.9%)	31(33.3%)	
** **segmental bronchus	11(13.8%)	4(4.4%)	0	11(11.8%)	
** **lobar bronchus	6(7.5%)	1(1.0%)	1(3.4%)	2(2.2%)	
Psammoma body	44(56.4%)	10(11.0%)	1(3.4%)	11(11.8%)	***p*** **<0.001**
Cholesterol cleft	32(40.5%)	12(13.2%)	2(6.9%)	8(8.6%)	***p*** **<0.001**
Pleural invasion	36(45%)	31(34.1%)	11(27.9%)	34(36.5%)	*p*>0.05
Vascular invasion	35(43.8%)	21(23.1%)	8(27.6%)	32(34.4%)	***p*** ** = 0.033**
Lymphatic invasion	49(62.0%)	31(40.4%)	11(37.9%)	42(45.2%)	***p*** ** = 0.047**
**Histologic subtypes**					
Predominant subtype					
** **lepidic	0	6(6.6%)	4(13.8%)	6(6.4%)	***p*** ** = 0.003**
** **papillary	17(21.3%)	22(24.2%)	3(10.3%)	19(20.4%)	*p*>0.05
** **acinar	23(28.7%)	53(58.2%)	17(58.6%)	38(40.9%)	***p*** **<0.001**
** **solid	35(43.8%)	5(5.5%)	5(17.2%)	17(18.3%)	***p*** **<0.001**
** **micropapillary	2(2.5%)	3(3.3%)	0	8(8.6%)	*p*>0.05
** **invasive mucinous	3(3.7%)	2(2.2%)	0	5(5.4%)	*p*>0.05
5% of subtype present					
** **lepidic	10(12.5%)	52(57.1%)	13(44.8%)	28(30.1%)	***p*** **<0.001**
** **papillary	31(38.8%)	46(50.5%)	10(34.5%)	42(45.2%)	*p*>0.05
** **acinar	42(53.8%)	82(90.1%)	24(82.8%)	69(74.2%)	***p*** **<0.001**
** **solid	54(67.5%)	18(19.8%)	11(37.9%)	25(26.9%)	***p*** **<0.001**
** **micropapillary	12(15.0%)	13(14.3%)	8(27.6%)	21(22.6%)	*p*>0.05
** **invasive mucinous	3(2.5%)	2(2.2%)	2(6.9%)	7(7.5%)	*p*>0.05

Abbreviations: TN: triple negative.

### Immunohistochemical Findings

#### Correlation between molecular subtype and immunohistochemical features of lung adenocarcinoma

**Figure 3:**
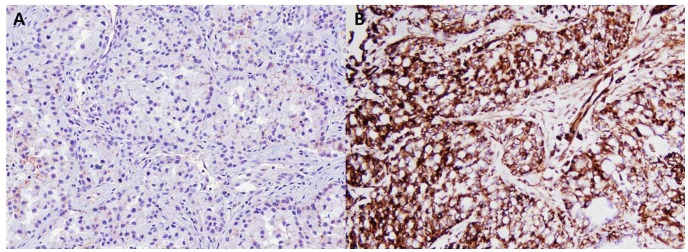
Expression of epithelial mesenchymal transition markers in *ALK*-rearranged tumors. A, Loss of E-cadherin; B, Expression of vimentin.

Several molecular markers were evaluated to investigate the origin of *ALK*-rearranged tumors. Type II pneumocytes served as a positive control for the expression of MUC-1 and SPs. A high positive rate of MUC-1, SP-A, and SP-B expression was present in all subgroups. The positive rate of MUC-1 and SP immunostaining was not significantly different between *ALK*-rearranged tumors and *ALK-*negative tumors (Table 4). TTF-1 positivity was more frequently observed in *EGFR*-mutated tumors (100%) than in *ALK*-rearranged (70%), *K-ras*-mutated (69%), and TN (70.8%) tumors (*p* = 0.001). In contrast, p63 immunostaining was significantly higher in *ALK*-rearranged tumors than in *ALK*-negative tumors (67.1%, 4.3%, 14.3%, and 14.6% of *ALK*-rearranged, *EGFR*-mutated, *K-ras*-mutated, and TN tumors, respectively; *p*<0.001). p40 positivity was observed in low frequency in all subgroups (2.9%, 4.3%, 7.1%, and 4.2% of *ALK*-rearranged, *EGFR*-mutated, *K-ras*-mutated, and TN tumors, respectively; *p*>0.05). Combined loss of E-cadherin and expression of vimentin, a representative marker of epithelial mesenchymal transition (EMT), was more commonly observed in *ALK*-rearranged tumors than other genotypes (38.9%, 19.1%, 26.9% and 14.6% of *ALK*-rearranged, *EGFR*-mutated, *K-ras*-mutated, and TN tumors, respectively; *p* = 0.015; Table 4; Figure 3).

**Table 4:** Immunohistochemical results for based on driver mutation status.

	*ALK*+(n = 80)	*EGFR*+(n = 91)	*K-ras*+(n = 29)	TN(n = 93)	*p* value
**MUC-1+**	98.70%	97.90%	96.30%	95.80%	*p*>0.05
**SP-A+**	73.70%	63.80%	59.30%	61.40%	*p*>0.05
**SP-B+**	61.50%	60.50%	59.30%	61.40%	*p*>0.05
**SP-C+**	25.30%	42.60%	28.60%	39.60%	*p*>0.05
**TTF-1+**	70.00%	100%	69.00%	70.80%	***p*** ** = 0.001**
**p63+**	67.10%	4.30%	14.30%	14.60%	***p*** **<0.001**
**p40**	2.90%	4.30%	7.10%	4.20%	*p*>0.05
**E-cadherin−**	71.30%	29.80%	55.20%	47.90%	***p*** **<0.001**
**Vimentin+**	49.30%	31.90%	37.00%	27.10%	*p* = 0.063
**EMT phenotype** *	38.90%	19.10%	26.90%	14.60%	***p*** ** = 0.015**

* loss of E-cadherin and expression of vimentin Abbreviations: TN: triple negative; SP: surfactant protein; TTF-1: Thyroid transcription factor 1.

### Multivariate Analysis

Results of the multivariate analysis are shown in Table 5. *ALK*-rearranged lung adenocarcinoma was significantly associated with the following morphological characteristics: cribriform formation (odds ratio [OR], 3.253; *p* = 0.028), presence of mucin-containing cells (OR, 4.899; *p* = 0.008), close relationship to adjacent bronchioles (OR, 5.361; *p* = 0.001), and presence of psammoma bodies (OR, 4.026; *p* = 0.002). *ALK*-rearranged tumors were also significantly associated with solid predominant histological subtype (OR, 13.685; *p* = 0.023).

**Table 5:** Multivariate analysis: Factors significantly associated with *ALK* rearrangement on logistic regression analysis.

	Odds ratio	*p* value	95% CI
Cribriform formation	3.253	**0.028**	1.133–9.341
Presence of extracellular mucin	0.775	0.689	0.223–2.691
Presence of mucin-containing cells	4.899	**0.008**	1.521–15.779
Close relation toadjacent bronchioles	5.361	**0.001**	2.032–14.149
Presence of psammoma body	4.026	**0.002**	1.633–9.930
Presence of cholesterol cleft	2.09	0.146	0.773–5.649
Solid predominant pattern	13.685	**0.023**	1.431–130.853

## Discussion

In this study, we performed a detailed comprehensive analysis of the histomorphological features associated with *ALK*-rearranged lung adenocarcinoma based on comparisons with well-known driver oncogene mutations. We found that *ALK*-rearranged tumors were significantly associated with younger age at presentation, frequent nodal metastasis, and higher stage of disease at diagnosis. Furthermore, *ALK*-rearranged lung adenocarcinoma exhibited several histological characteristics that differentiated it from other genotypes: cribriform formation, presence of mucin-containing cells, close relationship to adjacent bronchioles, presence of psammoma bodies, and solid predominant histological subtype. Correlation of histological characteristics with molecular alterations in lung adenocarcinoma may provide a new approach to refine pathological classification and its clinical relevance. To the best of our knowledge, this is the largest comprehensive analysis comparing the histomorphological features of resected *ALK-*rearranged tumors with other genotypes.

Our results revealed that the predominant histological subtype varied according to the status of driver mutations in *ALK*, *EGFR*, and *K-ras*. In contrast to *ALK*-negative tumors, *ALK*-rearranged tumors were significantly associated with solid predominant subtype and not acinar or papillary predominant subtypes. *ALK*-rearranged tumors exhibited aggressive behavior such as nodal metastasis, advanced disease stage at diagnosis, and lymphovascular invasion. Further, loss of E-cadherin and expression of vimentin, representing EMT phenotype, was frequently observed in *ALK*-rearranged tumors. EMT phenotype is the characteristic finding of *ALK*-rearranged tumors compared with other genotypes, and this could potentially be a contributing feature to the frequent metastases and high tumor stage seen in *ALK*-rearranged tumors. In contrast, *EGFR*-mutated tumors were significantly associated with acinar or papillary predominant subtypes. Although the lepidic predominant subtype was not significantly correlated with *EGFR* mutation, a significant correlation between lepidic component presence and *EGFR* mutation was observed. *EGFR*-mutated tumors have been shown to exhibit nonaggressive behavior, such as decreased nodal metastasis, lymphovascular invasion, and EMT feature. In the present study, the frequency of acinar and solid predominant patterns was higher in *K-ras*-mutated tumors than micropapillary and lepidic predominant patterns; however, it is difficult to assess the pathological relevance of these findings because of the small number of *K-ras*-mutated tumors. TN tumors were associated with acinar, papillary, and solid predominant patterns. Together our findings indicate that there is a strong association between genetic status and histological type based on the new IASLC/ATS/ERS lung adenocarcinoma classification.

Histomorphological features specific to *ALK*-rearranged tumors have been reported, including cribriform formation and the presence of mucin or mucin-containing cells and psammoma bodies. [16], [19] We also found that these features were strongly associated with *ALK-*rearranged tumors. We also identified that a close relationship to the adjacent bronchial epithelium is a unique feature of *ALK-*rearranged tumors. This close relationship with the bronchus was observed in 86.3% of *ALK*-rearranged tumors. Mucin containing tumor cells resembling the non-neoplastic goblet cells in segmental bronchus or bronchiole level were observed, and several tumor glands resembled the adjacent bronchial gland in morphology. In a few *ALK*-rearranged cases, tumor cells invaded the adjacent bronchiolar epithelium and showed the appearance of “budding off” of small epithelial cell clusters into the lumen. Furthermore, flat atypical lesions that resembled adjacent tumor cells infiltrated the non-neoplastic bronchial epithelium. *ALK*-rearranged tumors were more likely to be centrally located and easily obtained from the bronchoscopic biopsy procedure. Our findings suggest that *ALK*-rearranged tumors, in contrast to *EGFR*-mutated tumors, may represent non-TRU-type adenocarcinoma and therefore originate from bronchial epithelial cells. However, the expression of MUC-1, SP-A, SP-B, and SP-C was not different between *ALK*-rearranged and *ALK*-negative tumors. In contrast, TTF-1 and p63 expression was significantly different between *ALK*-rearranged and *ALK*-negative tumors, especially *EGFR*-mutated tumors. TTF-1 positivity was lower in *ALK*-rearranged tumors than in *EGFR*-mutated tumors, whereas p63 positivity was higher in *ALK*-rearranged tumors than in *EGFR*-mutated tumors. Although the frequency of TTF-1 positivity in *ALK*-rearranged tumors suggested they were of TRU-type origin, type II pneumocytes and Clara cells, which are characteristic of TRU-type, are typically negative for p63. [14] Our results suggested that a different mechanism mediates the development of *ALK*-rearranged tumors. We also evaluated the expression of p40 (△Np63) protein by immunohistochemistry. In contrast to p63, p40 positivity was less frequently observed in *ALK*-rearranged tumors (2.9%). p63 and p40 have been shown to be overexpressed especially in squamous cell carcinoma of lung and regarded as a marker of squamous differentiation. [27]–[31] However, several studies reported that p63 expression was seen in variable frequency (up to 30%) and extent (10 to 70% of tumor cells) in lung adenocarcinoma, whereas p40 was rarely expressed in adenocarcinoma. [32] In addition, Sakai et al. reported 7 out of 9 *ALK*-rearranged tumors expressed p63, but none of *ALK*-rearranged tumors expressed p40. [33] Our results are similar to those of these studies. We suggested that overexpression of p63 might have functional roles related with carcinogenesis or tumor differentiation rather than squamous markers in lung adenocarcinomas, but further investigation of the significance of p63 expression in lung adenocarcinoma is warranted. In the diagnostic point of view, spatial proximity between bronchus and *ALK*-rearranged tumors and frequent solid histologic subtype with p63 expression may cause diagnostic difficulties to differentiate squamous cell carcinoma in the small biopsy, whereas p40 was rarely expressed in *ALK*-rearranged adenocarcinoma. Awareness of these features may help pathologists diagnose accurately.

In conclusion, *ALK*-rearranged lung adenocarcinoma exhibited distinct clinicopathological and morphological features compared with other genotypes. Knowledge of these features may improve the diagnostic accuracy and lead to a better understanding of the characteristic behavior of *ALK*-rearranged tumors.
